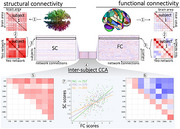# Structure‐function decoupling in genetic frontotemporal dementia

**DOI:** 10.1002/alz70856_100438

**Published:** 2025-12-25

**Authors:** Kamen A Tsvetanov, P Simon Jones, Maura Malpetti, Timothy Rittman, Arabella Bouzigues, John van Swieten, Lize Jiskoot, Harro Seelaar, Barbara Borroni, Enrico Premi, Raquel Sanchez‐Valle, Fermin Moreno, Robert Laforce, Caroline Graff, Matthis Synofzik, Daniela Galimberti, Mario Masellis, Carmela Tartaglia, Elizabeth Finger, Rik Vandenberghe, Alexandre de Mendonça, Fabrizio Tagliavini, Isabel Santana, Simon Ducharme, Christopher Butler, Alexander Gerhard, Johannes Levin, Markus Otto, Sandro Sorbi, Lucy L. Russell, Jonathan D. Rohrer, James B Rowe, the Genetic FTD Initiative, GENFI

**Affiliations:** ^1^ Department of Clinical Neurosciences, University of Cambridge, Cambridge, Cambridgeshire, United Kingdom; ^2^ Department of Psychology, University of Cambridge, Cambridge, United Kingdom; ^3^ Dementia Research Centre, Department of Neurodegenerative Disease, UCL Queen Square Institute of Neurology, University College London, London, London, United Kingdom; ^4^ Department of Neurology and Alzheimer Center, Erasmus Medical Center, Rotterdam, South Holland, Netherlands; ^5^ Erasmus MC University Medical Center, Rotterdam, Rotterdam, Netherlands; ^6^ Department of Neurology and Alzheimer Center, Erasmus Medical Center, Rotterdam, Zuid Holland, Netherlands; ^7^ University of Brescia, Brescia, Lombardy, Italy; ^8^ University of Brescia, Brescia, Italy; ^9^ Alzheimer's Disease and Other Cognitive Disorders Unit, Hospital Clínic, Institut d’Investigacions Biomediques August Pi i Sunyer (IDIBAPS), Barcelona, Spain; ^10^ Cognitive Disorders Unit, Department of Neurology, Donostia Universitary Hospital, San Sebastian, Spain; ^11^ Clinique Interdisciplinaire de mémoire, CHU de Québec ‐ Université Laval, Quebec City, QC, Canada; ^12^ Karolinska Institutet, Department NVS, Division of Neurogeriatrics, Stockholm, Sweden; ^13^ Department of Neurodegenerative Diseases, Hertie‐Institute for Clinical Brain Research and Center of Neurology, University of Tübingen, Tübingen, Germany; ^14^ German Center for Neurodegenerative Diseases (DZNE), University of Tubingen, Tubingen, Germany; ^15^ University of Milan, Milan, MI, Italy; ^16^ Fondazione IRCCS Ca’ Granda, Ospedale Policlinico, Neurodegenerative Diseases Unit, Milan, Italy; ^17^ LC Campbell Cognitive Neurology Research Unit, Sunnybrook Research Institute, Toronto, ON, Canada; ^18^ Toronto Western Hospital, Tanz Centre for Research in Neurodegenerative Disease, Toronto, ON, Canada; ^19^ University of Western Ontario, London, ON, Canada; ^20^ University Hospitals Leuven, Leuven, Belgium; ^21^ Laboratory for Cognitive Neurology, KU Leuven, Leuven, Leuven, Belgium; ^22^ Faculty of Medicine, University of Lisbon, Lisbon, Portugal; ^23^ Fondazione Istituto di Ricovero e Cura a Carattere Scientifico, Istituto Neurologica Carlo Besta, Milan, ‐, Italy; ^24^ Center for Neuroscience and Cell Biology, Faculty of Medicine, University of Coimbra, Coimbra, Coimbra, Portugal; ^25^ Neurology Department, Centro Hospitalar e Universitário de Coimbra, Coimbra, ‐, Portugal; ^26^ Department of Psychiatry, McGill University, Montreal, QC, Canada; ^27^ McConnell Brain Imaging Centre, Montreal Neurological Institute, McGill University, Montreal, QC, Canada; ^28^ University of Oxford, Oxford, United Kingdom; ^29^ Division of Neuroscience and Experimental Psychology, Wolfson Molecular Imaging Centre, University of Manchester, Manchester, ‐, United Kingdom; ^30^ Department of Geriatric Medicine, Klinikum Hochsauerland, Arnsberg, Arnsberg, Germany; ^31^ Neurologische Klinik und Poliklinik, Ludwig‐Maximilians‐Universität, Munich; German Center for Neurodegenerative Diseases (DZNE), Munich; Munich Cluster of Systems Neurology, Munich, Germany; ^32^ University Hospital Ulm, Ulm, ‐, Germany; ^33^ IRCCS Fondazione Don Carlo Gnocchi, Florence, Florence, Italy; ^34^ Department of Neuroscience, Psychology, Drug Research and Child Health, University of Florence, Florence, Italy; ^35^ MRC Cognition and Brain Sciences Unit, University of Cambridge, Cambridge, United Kingdom

## Abstract

**Background:**

Functional network integrity is important for maintaining cognitive performance during the 10‐20 year presymptomatic period of frontotemporal dementia (FTD), conferring resilience to advancing neuropathology and atrophy. The extent to which functional integrity relies on preserved structural connectivity is unclear. Here, we test the relationship between functional connectivity and structural connectivity, termed structure‐function coupling, against genetic risk for FTD and disease progression.

**Method:**

We studied 56 symptomatic and 165 pre‐symptomatic FTD‐mutation carriers, and 141 family members without mutations, from the GENFI cohort. Diffusion weighted imaging and functional magnetic resonance imaging (Siemens MR platforms) were acquired and analysed using established approaches to quantify participant‐level structural and functional connectomes (Figure 1‐(1)). Connectomes were defined in the Brainnetome Atlas and re‐mapped onto a subcortical network and seven resting‐state networks based on the Yeo Networks (Figure 1‐(2)). An inter‐subject regularized canonical correlation analysis (CCA) with permutation‐based cross‐validation was used to jointly analyse the structural and functional connectomes (Figure 1‐(3‐4)). Second‐level analysis with robust multiple linear regression models tested for differences between non‐carriers, pre‐symptomatic carriers and symptomatic carriers in the strength of association between structural and functional CCA subject scores. Age, sex, head motion and scanner site were included as covariates.

**Result:**

Canonical correlation analysis identified significant components linking structural and functional connectivity. The first component (r=0.656, *p* <0.001) reflected a structural connectivity pattern with high within‐ and between‐networks loadings (Figure 1‐(5)) with strong within‐networks functional connectivity and weak‐to‐negative between‐network functional connectivity (Figure 1‐(6)). This component associated structural integrity with function segregation, whereby individuals with high structural connectivity within and between networks exhibit greater functional network segregation as shown by strong within‐network functional connectivity and weak between network connectivity. The strength of this structure‐function coupling was greater for non‐carriers compared to pre‐symptomatic carriers (Figure 1‐(7)). Symptomatic carriers showed minimal relationship between structural and functional scores, indicating structure‐function decoupling, consistent with the hypothesis that cognitive decline is triggered by critical decoupling of previously synergistic neural systems.

**Conclusion:**

Our findings demonstrate progressive de‐coupling between structural connectivity and functional segregation over the course of genetic frontotemporal dementia. These results have implications for designing pre‐symptomatic disease‐modifying ‘preventative’ trials, supported by imaging‐based surrogate markers of neural system dynamics.